# Association between Tobacco Industry Interference Index (TIII) and MPOWER measures and adult daily smoking prevalence rate in 30 countries

**DOI:** 10.1186/s12992-023-01003-x

**Published:** 2024-01-03

**Authors:** Yuri Lee, Siwoo Kim, Min Kyung Kim, Ichiro Kawachi, Juhwan Oh

**Affiliations:** 1 Department of Health and Medical Information, Myongji College, Seoul, Republic of Korea; 2 Institute of Environmental Medicine, SNU Medical Research Center, 103 Daehakro, Seoul, Republic of Korea; 3 Epidemiology, Gillings School of Global Public Health, University of North Carolina at Chapel Hill, Chapel Hill, USA; 4 John L. Loeb & Frances Lehman Loeb Professor of Social Epidemiology, Department of Social and Behavioral Sciences, Harvard School of Public Health, 677 Huntington Ave., 7th floor, Boston, MA 02115 USA; 5 Department of Medicine, Seoul National University College of Medicine, 103 Daehakro, Seoul, Republic of Korea

**Keywords:** Tobacco Industry, Tobacco Policy, Smoking prevalence, FCTC, MPOWER

## Abstract

**Background:**

This study aimed to investigate the impact of tobacco industry interference on the implementation and management of tobacco control and the tobacco epidemic using the Tobacco Industry Interference Index (TIII) and MPOWER—a package of measures for tobacco control—and adult daily smoking prevalence in 30 countries.

**Methods:**

The TIII was extracted from the Global Tobacco Industry Interference Index 2019 and Global Center for Good Governance in Tobacco Control (GGTC). MPOWER measures and adult daily smoking prevalence rate were extracted from the World Health Organization (WHO) report on the global tobacco epidemic in 2021. We assessed the ecological cross-lagged association between TIII and MPOWER scores and between TIII and age-standardized prevalence rates for adult daily tobacco users.

**Results:**

Tobacco industry interference was inversely correlated with a country’s package of tobacco control measures (β = -0.088, *P* = 0.035). The TIII was correlated with weaker warnings about the dangers of tobacco (β = -0.016, *P* = 0.078) and lack of enforcement of bans on tobacco advertising promotion and sponsorship (β = -0.023, *P* = 0.026). In turn, the higher the TIII, the higher the age-standardized prevalence of adult daily tobacco smokers for both sexes (β = 0.170, *P* = 0.036). Adult daily smoking prevalence in males (β = 0.417, *P* = 0.004) was higher in countries where the tobacco industry received incentives that benefited its business.

**Conclusion:**

Where the interference of the tobacco industries was high, national compliance with the Framework Convention on Tobacco Control (FCTC) was lower, and the prevalence of adult daily smokers higher. National governments and global society must work together to minimize the tobacco industry’s efforts to interfere with tobacco control policies.

**Supplementary Information:**

The online version contains supplementary material available at 10.1186/s12992-023-01003-x.

## Introduction

Tobacco kills more than 8 million people annually. Despite the decline in tobacco smoking exposure in the last decade, tobacco remains the third leading risk factor for attributable disability-adjusted life-years (DALYs) among Level 2 risks defined by the Global Burden of Disease Study [1]. The tobacco industry has long operations and has used a range of methods to subvert the implementation of public health policies to combat the tobacco epidemic. Meanwhile, the tobacco industry has relentlessly promoted tobacco sales, despite knowing that tobacco damages people’s health for decades. The tobacco companies have been blamed for blocking, delaying, and weakening national tobacco control policies and World Health Organization (WHO) Framework Convention on Tobacco Control (FCTC, which was adopted in 2005. The Guidelines for implementation of Article 5.3, which was approved in 2008 [2], require FCTC Parties to protect public health policies from commercial and other vested interests of the tobacco industry.

In 2008, the WHO identified a package of measures under the acronym of MPOWER for reducing global morbidity and mortality with tobacco use through monitoring the implementation of the WHO FCTC and national tobacco control policies such as taxation, indoor smoking restrictions, contents regulation and disclosure, packaging and labeling, public awareness, banning promotion and sponsorship, prohibiting sales to minors and illicit trade, etc. [3]. The MPOWER policy package has been effective in reducing cigarette smoking among adults [4]. Husain et al. found that countries with higher tobacco control preparedness showed a significant reduction in daily adult smoking prevalence [5]. Regarding the association between progress in MPOWER implementation from 2008 to 2016 and smoking prevalence from 2009 to 2017, a unit increase of the MPOWER Score was associated with a 0.39 and 0.50 percentage points decrease in adult daily smoking prevalence in High MPOWER/high prevalence and High MPOWER/low prevalence countries, respectively [5]. Additionally, the WHO FCTC acknowledged the effect of diverse factors—trade liberalization, foreign direct investment, global marketing, transnational tobacco advertising, promotion, and sponsorship [6] – as facilitators in spreading the tobacco epidemic with cross-border effects [7]. Recently, tobacco industries have taken the Covid-19 pandemic as an opportunity to participate in relief efforts by donating ventilators, personal protective equipment, and cash to low- and middle-income countries to project a corporate image of social responsibility [8].

For many years, the tobacco industry has used various strategies to undermine the development, adoption, and implementation of public health policies aimed at addressing the tobacco epidemic. These strategies are shared across unhealthy commodity-producing industries such as sugar-sweetened beverages, ultra-processed likefood and alcohol [9]. Tobacco industry interference has taken many forms, including: 1) influencing the political and legislative process through lobbying and political donations; 2) exaggerating the economic importance of the industry, often using threats of job losses, which is especially effective in countries with high unemployment rates; 3) manipulating public opinion; 4) fabricating support through front groups; 5) discrediting proven science, and 6) intimidating governments with litigation [9, 10].

No studies have empirically examined how successfully the tobacco industry has attempted to interfere with implementing tobacco control policies. To address this gap, this study includes three aims: 1) to explore the association between the Tobacco Industry Inference Index (TIII) and MPOWER scores, as well as the association between TIII and adult daily smoking prevalence across 30 countries; 2) to investigate the association between the subcomponents of the TIII and the subcomponents of the MPOWER package; and 3) to explore the various types of TIII and the gender stratified adult daily smoking prevalence.

## Methods

### Data sources

The TIII was obtained from the Global Center for Good Governance in Tobacco Control (GGTC), which collects and publishes information. The index released in 2019 included 33 countries from Africa, the Eastern Mediterranean region, Latin and North America, Europe, Asia, and the Western Pacific region. After excluding missing data, 30 countries were analyzed. The TIII is based on publicly available information on tobacco industry interference in countries and their respective governments’ responses to this interference in implementing the WHO FCTC Article 5.3 and its Guidelines from January 2017 to December 2018 [11]. In 2023, the GGTC updated its coverage to encompass 80 countries. After excluding missing data, analysis included 72 countries (results are presented in the supplementary material). This 2023 GGTC dataset also includes all the 30 countries of the 2019 GGTC dataset.

The MPOWER scores were obtained from the WHO report on the Global Tobacco Epidemic 2021 based on surveys completed in 2018 and 2019. The latest data on adult daily smoking prevalence from 2019 was sourced from the 2021 WHO report summarizing the age-standardized prevalence rates for adult daily tobacco smokers in both sexes [12].

### Variables

The TIII (range 0–100) estimates the response of governments to tobacco industry interference and their public health policies from vested interests as required under the WHO FCTC. Thus, the higher the TIII score, the higher the overall level of tobacco industry interference. For example, the TIII score of the Dominican Republic is 96, indicating a very active and robust tobacco industry interference. In contrast, Brunei Darussalam scored the lowest of 15, showing a solid government power to regulate the tobacco industries.

The TIII includes seven subcomponents: 1) ‘participation in policy development’ which means the tobacco industry interfered in tobacco control public policy generation and implementation (4 categories, 0–20 scores); 2) ‘tobacco-related corporate social responsibility (CSR) activities’ which means the government receives contributions from the tobacco industry or participates in tobacco industry CSR activities (1 category, 0–5 scores); 3) ‘benefits to the tobacco industry’ which means the tobacco industry received incentives that benefited its business (2 categories, 0–10 scores); 4) ‘forms of unnecessary interaction’ which means inappropriate interactions occurred between governments and the industry (3 categories, 0–15 scores); 5) (level of non-) ‘transparency’ which means the government does not publicly disclose interactions with the tobacco industry (2 categories, 0–10 scores); and 6) ‘conflict of interest’ which means that public officials faced conflicts of interest situations (3 categories, 0–15 scores), and 7) ‘preventive measures’ which mean that governments have acted to protect themselves with preventive measures (5 categories, 0–25 scores, reverse coding).

The MPOWER package comprises evidence-based tobacco control measures, including six proven policies. M contains monitoring tobacco use and preventive measures and five attainment levels for each other components [5]. P means protecting the population from cigarette smoke, such as designing non-smoking areas in public places. O indicates help to quit tobacco use, such as smoking cessation clinics or cessation call centers. W consists of packaging a health warning about the dangers of tobacco (W1) and implementing a non-smoking campaign for cigarette risk (W2). E summarizes advertising restrictions on tobacco promotion and sponsorship. R means raising taxes on tobacco.

The M policy dimension ranges from 1 (lowest) to 4 (highest) attainment level for scoring. All other POWER policy dimension, the score ranges from 1 (weakest) to 5 (strongest) policies. Since there are seven MPOWER categories, the minimum MPOWER score is 7, and the maximum achievable MPOWER score is 4 + (5 × 6) or 34 [12]. A high MPOWER score indicates strong tobacco control in the country [13, 14]. We used the same manner as the latest study [5] using MPOWER Scores by excluding R (the taxation component), which ranges from a minimum of 6 to a maximum of 29 in our analysis: MPOWE(R). Aggregated MPOWER scores help to generate a consistent ranking of implementation status across countries and over time.

Adult daily smoking prevalence is age-standardized prevalence rates for adult daily smokers (aged 15 years or older) of tobacco in both sexes, which was constructed to compare tobacco use prevalence estimates across multiple countries or various periods of the same country. Daily cigarette smoking use varies between surveys but usually measures cigarette use at least once daily.

### Statistical analysis

Three analyses were performed using a nation as a unit of analysis: Ecological analysis and a cross-lagged panel model were applied. First, co-adjusted multiple regressions were performed on the association between the TIII and MPOWER scores and TIII and adult daily smoking prevalence. Second, decomposition regression analyses were measured to understand the MPOWER package score and seven subcomponents of MPOWER measures. Third, gender-stratified regression analysis between decomposed TIII and adult daily smoking prevalence was conducted. Statistical significance was determined as a *p*-value of < 0.05 in a two-sided manner. All statistical analysis was performed with SPSS 25.0 (SPSS et al., USA).

### Ethical statement

This study was exempt from institutional review board approval because we used publicly available data containing no personal identifiers.

## Results

**Fig. 1:**
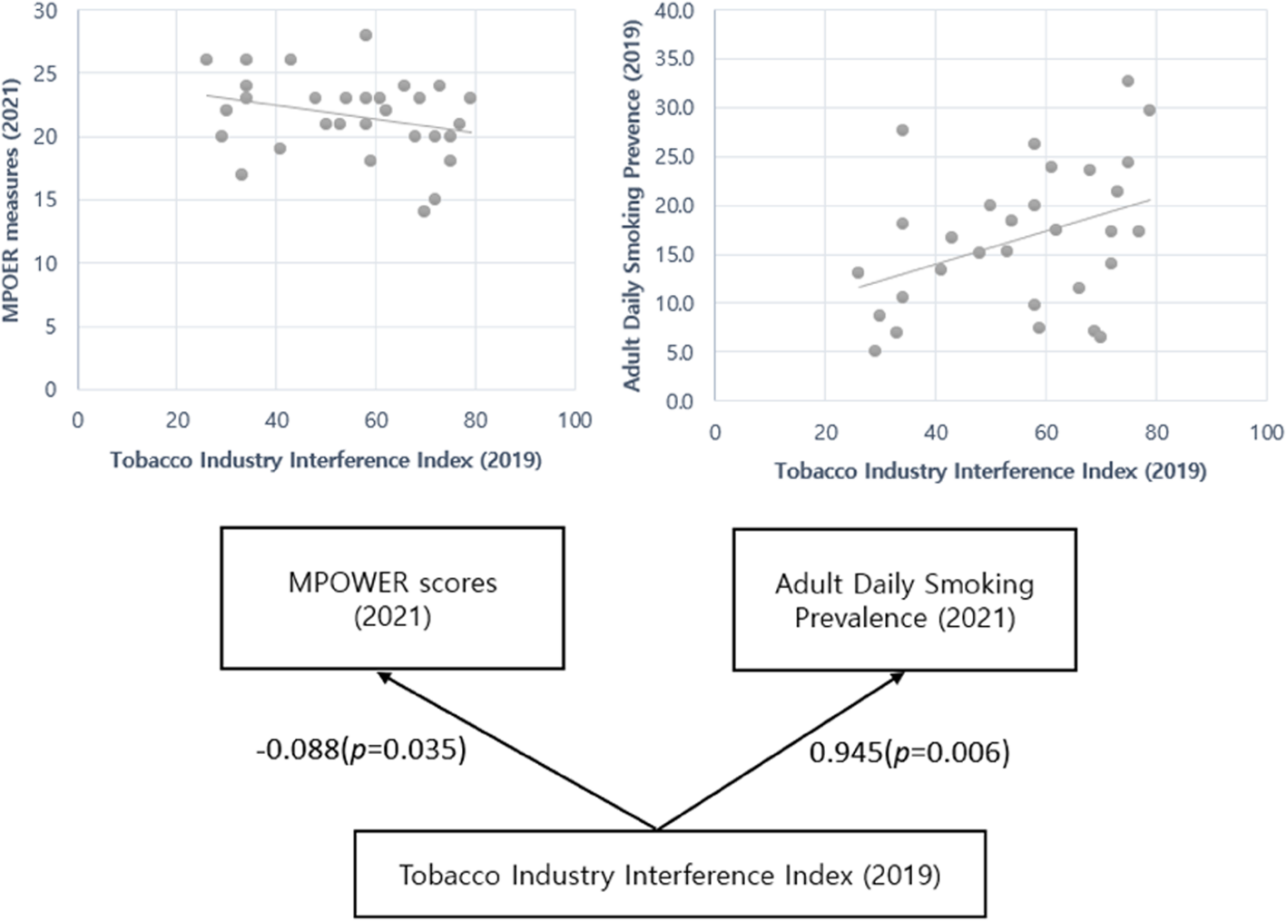
Cross-lagged Association between Tobacco Industry Interference Index (TIII) and MPOWER scores and adult daily smoking prevalence in 30 countries. Note: Adjusted adult daily smoking prevalence or MPOWER scores. *P* < 0.05*

The unadjusted regression analysis revealed a clear inverse relationship between the TIII and MPOWER scores (β = -0.088, *P* = 0.035). In contrast, the TIII showed a marginally positive association with age-standardized prevalence rates for adult daily tobacco smokers in both sexes (β = 0.945, *P* = 0.006) after mutual statistical adjustment (Fig. 1). Cross-sectional association between TIII (2021) and the MPOWER scores (β = -0.083, *P* = 0.004) and adult daily smoking prevalence (2021) (β = 0.162, *P* = 0.083) in 72 countries showed similar results (Figure S1, Table S1-5). There was no statistical correlation between the TIII and MPOWER scores in countries with state-owned tobacco companies (SOTCs) and in Lebanon and Japan, where the governments are the significant stakeholders of tobacco companies (β = -0.037, *P* = 0.617) (Table S2).

Among subcomponents of the TIII, as the prevention measures such as the government’s procedures for disclosing records of the interaction with the tobacco industry, programs to consistently raise awareness on policies relating to FCTC Article 5.3 Guidelines increased, the MPOWER package got lowered (β = -0.240, *P* = 0.035) (Table 1).

**Table 1:** Impact of the subcomponents of the Tobacco Industry Interference (TIII) on MPOWER scores in 30 countries

Variable	MPOWER Scores (2021)	
	β	*P*-value
Tobacco Industry Interference Index (2019)	-0.055	0.125
Participation in Policy Development	-0.186	0.121
Tobacco-related CSR Activities	-0.348	0.296
Benefits to the Tobacco Industry	-0.139	0.527
Forms of Unnecessary Interaction	-0.069	0.591
Conflict of Interest	-0.166	0.560
Transparency	0.027	0.882
Preventive Measures	-0.240*	0.035

*P* < 0.05*

The TIII was inversely associated with E (enforcing bans on tobacco advertising promotion and sponsorship, TAPS) (β = -0.023, *P* = 0.026), which means that the more vulnerable countries to the tobacco industry interference, the lower the degree of TAPS policies. Countries with a higher degree of tobacco interference were less likely to inform the public of the dangers of tobacco through health warning labels (β = -0.016, *P* = 0.078). The greater the tobacco industries’ tobacco-related CSR activities, the lower the enforcement of package warnings about the dangers of tobacco (β = -0.179, *P* = 0.030) (Table 2). On the other hand, the TIII showed no significant association with other MPOWER components such as M (monitor tobacco use and preventive policies), P (protect from tobacco smoke), O (offer help to quit tobacco use), and R (raise taxes on tobacco) (Table 2).

**Table 2:** Impact of the Subcomponents of the TIII on Subcomponents of the MPOWER package in 30 countries

Variable	M		*P*		O		W(1)		W(2)		E		R	
	β	P-value	β	*P*-value	β	*P*-value	β	*P*-value	β	*P*-value	β	*P*-value	β	*P*-value
Tobacco Industry Interference Index (2019)	-0.003	0.766	-0.019	0.193	0.003	0.793	-0.016	0.078	0.005	0.738	-0.023*	0.026	-0.002	0.854
Participation in Policy Development	-0.005	0.883	-0.081	0.086	0.015	0.630	-0.045	0.140	0.035	0.442	-0.067	0.051	-0.038	0.263
Tobacco-related CSR Activities	-0.079	0.382	-0.149	0.257	0.000	1.000	-0.179*	0.030	0.188	0.125	-0.111	0.250	-0.020	0.837
Benefits to the Tobacco Industry	0.051	0.381	-0.065	0.452	-0.036	0.529	-0.040	0.472	-0.011	0.892	-0.038	0.549	0.000	1.000
Forms of Unnecessary Interaction	-0.021	0.550	-0.007	0.892	0.014	0.680	-0.036	0.272	0.002	0.967	-0.036	0.323	0.015	0.681
Conflict of Interest	0.053	0.490	0.020	0.858	0.015	0.839-	-0.048	0.512	0.003	0.981	-0.146	0.070	-0.033	0.686
Transparency	-0.009	0.851	-0.023	0.754	0.046	0.326	0.007	0.881	0.031	0.647	-0.082	0.112	0.057	0.267
Preventive Measures	-0.019	0.552	-0.068	0.138	-0.007	0.832	-0.056	0.058	-0.021	0.640	-0.058	0.085	-0.013	0.707

*P* < 0.05*. M contains monitoring tobacco use and preventive measures and five attainment levels for each other components. P means protecting the population from cigarette smoke, such as designing non-smoking areas in public places. O indicates help to quit tobacco use, such as smoking cessation clinics or cessation call centers. W consists of packaging a health warning about the dangers of tobacco (W1) and implementing a non-smoking campaign for cigarette risk (W2). E summarizes advertising on tobacco ban, promotion and sponsorship. R means raising taxes on tobacco

In the gender stratified analysis, the greater the TIII, the higher age-standardized prevalence rates for adult daily smokers of tobacco among males (β = 0.417, *P* = 0.004), whereas there was no significant association among females (β = -0.062, *P* = 0.390). In the decomposed analysis looking at each component of the TIII, adult daily smoking prevalence in the male group was higher in countries where the tobacco industry received incentives in the form of business ‘benefits to the tobacco industries’ (β = 2.073, *P* = 0.036), the government receives contributions from the tobacco industry or participates in tobacco industry CSR activities ‘Tobacco-related CSR Activities’ (β = 3.099, *P* = 0.020), ‘Participation in Policy Development’ by tobacco industries (β = 0.463, *P* = 0.023), and inappropriate interactions occurred between governments and the industry ‘Forms of Unnecessary Interaction’ (β = 1.093, *P* = 0.036) (Table 3).

**Table 3:** Impact of the Subcomponents of the TIII on Adult Daily Smoking Prevalence by Gender in 30 countries

Variable	Adult daily smoking prevalence (2021)					
	Both Sexes		Male		Female	
	β	*P*-value	β	P-value	β	*P-*value
Tobacco Industry Interference Index (2019)	0.170*	0.036	0.417*	0.004	-0.062	0.390
Participation in Policy Development	0.513	0.060	0.463*	0.023	-0.040	0.868
Tobacco-related CSR Activities	0.772	0.314	3.099*	0.020	-1.534*	0.014
Benefits to the Tobacco Industry	0.944	0.053	2.073*	0.017	-0.170	0.691
Forms of Unnecessary Interaction	0.511	0.074	1.093*	0.036	0.005	0.985
Conflict of Interest	0.636	0.328	2.081	0.070	-0.806	0.141
Transparency	0.270	0.517	1.264	0.102	-0.688*	0.045
Preventive Measures	0.225	0.346	0.519	0.305	0.067	0.774

*P* < 0.05*

## Discussion

An inverse association between the TIII and MPOWER measures and a positive association between the TIII and adult daily smoking prevalence was found. The higher the benefit to tobacco industries or the fewer the preventive measures, the lower the country’s MPOWER package implementation. Countries with high tobacco industry interference had a low level of tobacco hazard warning policies or tobacco advertising prohibition policies. Where the tobacco industry received incentives that benefited its business and public officials faced conflicts of interest with tobacco companies, smoking prevalence was high in some countries. There was little effect in females for tobacco industry interference, and most adults daily smoking, but a positive effect was shown among males.

WHO Article 5.3 has guideline recommendations on awareness-raising, limiting interaction with the tobacco industry, rejection of industry partnerships, avoidance of conflicts of interest, transparency, denormalization of industry CSR activities, preferential treatment of the tobacco industry, and state-owned tobacco industries [14]. According to Fooks et al., only 6% of parties have introduced more than half of the requirements of Article 5.3. Moreover, 16% of guideline recommendations have been implemented among parties, implying an extensive opportunity for tobacco industry influence [15]. Parties must remain alert and ensure that Article 5.3 is implemented across different government sectors, aiming at policy coherence that does not favor the tobacco industry’s interests over parties’ obligations under the FCTC [2]. United States of America (USA), who has leading cigarette manufacturing, remains a non-ratified FCTC party [16].

The study results support the previous findings that the tobacco industry’s interference weakens the national tobacco control policy or MPOWER package [17]. Our results confirmed an argument that tobacco companies' strategies interfered with tobacco control policies which might contribute to increasing the smoking prevalence. When the government’s preventive measures against interference in the tobacco industry were insufficient, or when the tobacco industry benefited greatly, the implementation of FCTC and MPOWER scores were lower. In particular, the tobacco industry exerted a powerful force to block the policy of inserting warnings or pictures on tobacco packages or weaken the ban on sponsoring advertisements for tobacco products. This study identifies that several measures protecting tobacco control policies from the commercial and other vested interests of the tobacco industry have not yet completely prevented the tobacco industry from interfering with tobacco control policies [2]. The study results also show higher rates of male smoking in countries where public officials face conflicts of interest, consistent with Article 5.3 guidelines recommending rules for public servants to avoid conflicts of interest for protecting public health policies from interference with the tobacco industry [18]. Legislators have become vulnerable to tobacco business interference by receiving political contributions [19].

The study also found that the greater the tobacco interference, the lower level of warning about the dangers of tobacco which shows tobacco companies use packaging and other advertising techniques to make cigarettes attractive and misrepresent the fact that tobacco products are detrimental to health [20]. In addition, we found that enforcement of bans on TAPS decreased as tobacco business interference increased, contrary to Article 13 of the FCTC. We recommend that all tobacco industry interference with TAPS should be regulated, including direct advertising bans such as mass media, outdoor advertising, point-of-sale (POS) advertising materials, indirect advertising bans inducing brand stretching, brand sharing, and product placement, etc. [21].

This study also supports the previous hypothesis that countries with high tobacco industry interference have more problems with tobacco use or smoking epidemics [22]. The sabotage strategy from the tobacco industry weakens the MPOWER package and national tobacco control level. Our study results showed that tobacco industry’s activities increased the population's tobacco consumption, confirming that the tobacco industry might be a primary facilitator of the tobacco epidemic. Meanwhile, tobacco use can be explained as the tobacco user as a host, the tobacco product as an agent, and policy or media as an environment [23]. Eliminating the tobacco industry’s influence on health policy is vital for effective tobacco control and reducing health risks. The WHO underscores that raising tobacco taxes is the most effective strategy to curb tobacco use [14]. Considering the tobacco industry’s strong opposition to such tax measures, it’s evident that their role in health policy, especially in taxation matters, needs stringent oversight.

The results show that the tobacco industry's influence on the smoking prevalence rate is more significant for males than females. Given the smoking epidemic in women generally lagging behind men by 30–40 years [24], it is expected that smoking among women will increase first among those with higher socioeconomic status, as it did with men several decades prior, and a gap between gender will continue to narrow [25]. Historical women's liberation movements encouraging equal smoking to men might have increased the women’s smoking rate even in the low TIII countries might have nullified existing associations in women subgroup analysis. In this context, tobacco business activities could impact the increase in the smoking rate for both sexes.

One of the most vital indicators of conflict of interest is whether the government owns the tobacco companies through weak tobacco control in SOTCs, such as low tax rates, no indoor smoking restrictions, sales of tobacco products to youth, and loose advertising regulations, etc. [25] Countries in which the government monopolizes the production and sales of tobacco products or owns at least one tobacco company must have a high TIII score based on a conflict of interest [26]. Contrary to expectation, the TIII scores were not significantly higher in SOTCs. Based on economic theory, one possible reason might be that tobacco company monopolies lack competitive incentives to actively engage in sales tactics such as mass advertising and encouraging sales to youth and women. The other possible reason is the small sample size for countries with SOTCs.

Some limitations need to be considered when interpreting the results. First, our findings from the 30 selected countries may not generalize to a wider pool of countries. Second, measurement errors need to be considered. Random measurement error in the exposure variable will bias associations towards the null [27]. Previous studies have used TIII as an exposure variable. In a study that evaluated the degree of FCTC 5.3 implementation in seven Asian countries using the TIII, the scoring system was designed with the help of tobacco control experts and validated through a focused group discussion [28]. Hoe et al. have argued that one of the most cited barriers to tobacco control policy implementation worldwide is tobacco industry interference. At the same time, TIII 2019 showed that adherence to Article 5.3 had been far from satisfactory worldwide [29]. Jeffrey Drope et al. found strong evidence that interference in tobacco control policymaking has increased in some countries in Latin America with the assessment results using the TIII 2020 [30].

Brunei Darussalam's case offers a compelling insight into the complexities of tobacco control. Despite its regulatory solid stance, evidenced by a low score on the Tobacco Industry Interference Index, tobacco consumption has only slightly declined from 16.7% in 2000 to 16.2% in 2020 [31, 32]. This suggests that while regulations are vital, they might need to be more sufficient on their own. A holistic approach, combining regulations with public awareness campaigns, education, and community engagement, is essential for impactful tobacco control. Brunei's experience underscores the importance of ongoing monitoring and adaptable public health strategies. While the FCTC and MPOWER were introduced in 2005, tobacco policies existed long before that date. Countries already had diverse trajectories of smoking prevalence rates prior to the introduction of these initiatives. Our World in Data reveals variations in tobacco use trajectories following the FCTC enactment in 2005. Some countries saw tobacco use rise by over 30%, while others reported a decline of more than 50% [33]. Analyzing countries with increased smoking rates post-FCTC against those with significant declines could provide insights into the effectiveness of different TIII scores and broader influences on tobacco consumption trends. This comparative analysis can provide valuable insights into effectiveness of different TIII scores and the broader influences on tobacco consumption trends. However, there were too few countries in our data to permit a stratified analysis.

As the national compliance with the FCTC decreased, prevalence rates for adult daily tobacco smokers increased if the tobacco industries’ interference was high. Tobacco industry tactics have been evolving to promote and grow tobacco businesses. Thus, it is crucial to resist tobacco industry disturbances to protect public health and save lives from harmful tobacco use. Governments and global society should counter tobacco industry interference to protect public health and advance tobacco control policies.

### Supplementary Information

**Additional file 1:** **Figure S1.** Association between Tobacco Industry Interference Index (TIII) and MPOWER scores and adult daily smoking prevalence in 72 countries. **Table S1.** General Characteristics of the Countries (N=72**). Table S2.** Impact of the Tobacco Industry Interference (TIII) on MPOWER scores and adult daily smoking prevalence rate in 72 countries (Stratification by the State-Owned Tobacco Companies, SOTCs). **Table S3.** Impact of the subcomponents of the Tobacco Industry Interference (TIII) on MPOWER scores in 72 countries. **Table S4.** Impact of the Subcomponents of the TIII on Subcomponents of the MPOWER package in 72 countries. **Table S5.** Impact of the Subcomponents of the TIII on Adult Daily Smoking Prevalence by Gender in 72 countries.

## Data Availability

Publicly available data are used in the study. All data relevant to the study are provided with the sources in the article.

## Abbreviations

TIII: Tobacco industry interference index
GGTC: Global center for good governance in tobacco control
WHO: World health organization
FCTC: Framework convention on tobacco control
DALYs: Disability adjusted life years
SOTCs: State owned tobacco companies
TAPS: Tobacco advertising promotion and sponsorship
CSR: Corporate social responsibility

## References

[1] Murray CJ, Aravkin AY, Zheng P, Abbafati C, Abbas KM, Abbasi-Kangevari M, Borzouei S (2020). Global burden of 87 risk factors in 204 countries and territories, 1990–2019: a systematic analysis for the Global Burden of Disease Study 2019. Lancet.

[2] Bialous SA (2019). Impact of implementation of the WHO FCTC on the tobacco industry’s behaviour. Tob Control.

[3] World Health Organization. (2008). MPOWER: a policy package to reverse the tobacco epidemic. Geneva: World Health Organization. Available: https://apps.who.int/iris/handle/10665/43888 Accessed 20 May 2023.

[4] Dubray J, Schwartz R, Chaiton M, O’Connor S, Cohen JE (2015). The effect of MPOWER on smoking prevalence. Tob Control.

[5] Husain MJ, Datta BK, Nargis N, Iglesias R, Perucic AM, Ahluwalia IB, Richter P (2021). Revisiting the association between worldwide implementation of the MPOWER package and smoking prevalence, 2008–2017. Tobacco Control.

[6] Burci, G. L. World Health Organization (WHO): framework convention on tobacco control. International Legal Materials, 2003;42(3):515–539. Available: https://apps.who.int/iris/handle/10665/78302 Accessed 20 May 2023.

[7] Zhang K, Tartarone A, Pérez-Ríos M, Novello S, Mariniello A, Roviello G, Zhang J (2022). Smoking burden, MPOWER, future tobacco control and real-world challenges in China: reflections on the WHO report on the global tobacco epidemic 2021. Translational Lung Cancer Res.

[8] Yadav A, Lal P, Sharma R, Pandey A, Singh RJ (2021). Tobacco industry corporate social responsibility activities amid COVID-19 pandemic in India. Tob Control.

[9] Maani N, Petticrew M, Galea S (2023). The Commercial Determinants of Health.

[10] World Health Organization. Tobacco industry interference: A global brief (No. WHO/NMH/TFI/12.1). Geneva: World Health Organization; 2012.

[11] Assunta, M. (2019). Global tobacco industry interference index 2019. Available: https://exposetobacco.org/wp-content/uploads/2019/10/GlobalTIIIndex_Report_2019.pdf [Accessed 20 May 2023].

[12] World Health Organization. (2021). WHO Report on the Global Tobacco Epidemic, 2021: Addressing new and emerging products. World Health Organization. https://www.who.int/publications/i/item/9789240032095 Accessed 20 May 2023.

[13] Gezer, T., Dagli, E., Yildiz, F., Ay, P., Elbek, O., Ceyhan, M., & Güner, M. Why does tobacco consumption increase in a MPOWER-compliant country? Tobacco Induced Diseases, 2018;16(1). 10.18332/tid/84057

[14] Goel S, Kar SS, Verma M, Sivanantham P, Naik BN, Gupta D (2021). Evidence on article 5.3 of FCTC (tobacco industry interference in tobacco control activities) in India-a qualitative scoping study. BMC public health.

[15] Fooks GJ, Smith J, Lee K, Holden C (2017). Controlling corporate influence in health policy making? An assessment of the implementation of article 5.3 of the World Health Organization framework convention on tobacco control. Globalization and health.

[16] Lempert LK, Glantz SA (2018). Heated tobacco product regulation under US law and the FCTC. Tob Control.

[17] King BA, Ahluwalia IB, Gomes AB, Fong GT (2022). Combating the tobacco epidemic in North America: challenges and opportunities. Tob Control.

[18] Rao NV, Bhojani U, Shekar P, Daddi S (2016). In many countries Conflicts of interest in tobacco control in India: an exploratory study. Tobacco Control.

[19] Balwicki Ł, Stokłosa M, Balwicka-Szczyrba M, Tomczak W (2016). Tobacco industry interference with tobacco control policies in Poland: legal aspects and industry practices. Tob Control.

[20] Lempert LK, Glantz S (2017). Packaging colour research by tobacco companies: the pack as a product characteristic. Tob Control.

[21] Freeman, B., Watts, C., Astuti, P. A. S. Global tobacco advertising, promotion and sponsorship regulation: what’s old, what’s new and where to next? 2022;216–221. 10.1136/tobaccocontrol-2021-056551 10.1136/tobaccocontrol-2021-056551 35241591

[22] Sóñora G, Reynales-Shigematsu LM, Barnoya J, Llorente B, Szklo AS, Thrasher JF (2022). Achievements, challenges, priorities and needs to address the current tobacco epidemic in Latin America. Tob Control.

[23] Ribisl, K. M., Chaloupka, F. J., Kirchner, T. R., Henriksen, L., Nettles, D. S., Geisler, R. C., ... & Swan, G. E. (2020). PhenX: Vector measures for tobacco regulatory research. Tobacco control, 29(Suppl 1), s27-s34. 10.1136/tobaccocontrol-2019-054977 10.1136/tobaccocontrol-2019-054977 PMC7842080 31992661

[24] Janssen F (2021). The Role of Smoking in Country Differences in Life Expectancy Across Europe, 1985–2014. Nicotine Tob Res.

[25] Solomon A (2020). Gender, women, and the future of tobacco control. Drugs Alcohol Today.

[26] Hogg SL, Hill SE, Collin J (2016). State-ownership of tobacco industry: a ‘fundamental conflict of interest’ or a ‘tremendous opportunity’ for tobacco control?. Tob Control.

[27] Freedman LS, Schatzkin A, Midthune D, Kipnis V (2011). Dealing with dietary measurement error in nutritional cohort studies. J Natl Cancer Inst.

[28] Assunta M, Dorotheo EU (2016). SEATCA tobacco industry interference index: a tool for measuring implementation of WHO framework convention on tobacco control article 53. Tobacco control.

[29] Hoe C, Kennedy RD, Spires M, Tamplin S, Cohen JE (2019). Improving the implementation of tobacco control policies in low-and middle-income countries: a proposed framework. BMJ Glob Health.

[30] Drope J, Rodriguez-Iglesias G, Stoklosa M, Szklo A. Recent evidence on the illicit cigarette trade in Latin America. Revista Panamericana de Salud Pública, 46, Special Issue: Tobacco Control. 2022. 10.26633/rpsp.2022.111. 10.26633/RPSP.2022.111 PMC9534335 36211247

[31] World Health Organization. WHO global report on trends in prevalence of tobacco use 2000–2025 (3rd ed.). Geneva: World Health Organization; 2019.

[32] The World Bank. Prevalence of current tobacco use (% of adults) Brunei Darussalam. https://data.worldbank.org/indicator/SH.PRV.SMOK?locations=BN Accessed 17 Aug 2023.

[33] Hannah Ritchie and Max Roser (2013) - "Smoking". Published online at Our World In Data.org. Retrieved from: https://ourworldindata.org/smoking.

